# Elevated Thyroid Peroxidase Antibody Increases Risk of Post-partum Depression by Decreasing Prefrontal Cortex BDNF and 5-HT Levels in Mice

**DOI:** 10.3389/fncel.2016.00307

**Published:** 2017-01-09

**Authors:** Yingying Zhou, Xinyi Wang, Yuhang Zhao, Aihua Liu, Tong Zhao, Yuanyuan Zhang, Zhongyan Shan, Weiping Teng

**Affiliations:** ^1^Department of Endocrinology and Metabolism, Institute of Endocrinology, Liaoning Provincial Key Laboratory of Endocrine Diseases, The First Affiliated Hospital, China Medical UniversityShenyang, China; ^2^Department of Laboratory Medicine, The First Affiliated Hospital, China Medical UniversityShenyang, China; ^3^Department of Endocrinology, Affiliated Hospital of Qingdao UniversityQingdao, China

**Keywords:** thyroid peroxidase antibody (TPOAb), post-partum depression (PPD), thyroid hormone, brain-derived neurotrophic factor (BDNF), serotonin (5-HT)

## Abstract

Post-partum depression (PPD) is a common mental disease in the perinatal period that profoundly affects mothers and their offspring. Some clinical studies have found that PPD is related to thyroid peroxidase antibodies (TPOAbs); however, the mechanism underlying this relationship is unclear. Female C57BL/6 mice immunized with adenovirus encoding the cDNA of the full-length mTPO (mTPO-Ad) were used to establish the isolated TPOAb-positive mouse model in the present study. Maternal depressive-like behaviors were assessed using the forced swimming test (FST), sucrose preference test (SPT), and tail suspension test (TST) post-partum. The serum TPOAb titer was measured by enzyme-linked immunosorbent assay (ELISA) before pregnancy and post-partum. Furthermore, in the prefrontal cortex, the mRNA and protein expression levels of brain-derived neurotrophic factor (BDNF) were measured, serotonin (5-HT) levels were measured by ultra-high-performance liquid chromatography–tandem mass-spectrometry (UHPLC–MS/MS), and total thyroxine (TT4) levels were determined by ELISA. Compared with the controls, the mice immunized with mTPO-Ad displayed depressive behaviors, with a significantly lower sucrose preference (SP) at the 12-h time point and a longer immobility time in the FST and TST, which were accompanied by a lower expression of BDNF and 5-HT but no change in the TT4 concentration in the prefrontal cortex. Together, these findings suggest that elevated TPOAb may increase the risk of subsequent PPD and decrease the concentration of BDNF and 5-HT in the prefrontal cortex.

## Introduction

Elevated thyroid peroxidase antibodie (TPOAb), with or without thyroid dysfunction, is characteristic of autoimmune thyroiditis (AIT). Both AIT and mood disorders are prevalent and might even occur simultaneously. The prevalence of TPOAb or TgAb in an euthyroid status in pregnant women and women with post-partum depression (PPD) ranges from 10–20% (Stagnaro-Green et al., 2011) and 10–15% (Gaynes et al., 2005), respectively. Over the past few decades, clinical studies have focused on the association between TPOAb and depression. Of those, several reports have noted that TPOAb is linked to depression (Pop et al., 1998; Carta et al., 2004; Ott et al., 2011; Watt et al., 2012), and TPOAb has come to be regarded as a marker of vulnerability for depression (van de Ven et al., 2012). However, recent studies also came to the opposite conclusion: that there was no association between TPOAb and depressive symptoms (Delitala et al., 2016) and that TPOAb could not predict PPD (Albacar et al., 2010).

Emerging evidence implicates brain-derived neurotrophic factor (BDNF) and serotonin (5-HT) in the pathophysiology of depression and in the actions of antidepressant agents (Homberg et al., 2014; Mahar et al., 2014). BDNF is an important regulator of synaptic plasticity in the brain and provides neurotrophic support to diverse neuronal populations, including serotonergic neurons (Daftary et al., 2012). Furthermore, genetic and functional evidence suggests that there is a dysregulated interaction between BDNF signaling and serotonergic neurotransmission in depression pathology (Wells et al., 2010). The prefrontal cortex is involved in the regulation of mood and stress (Jia et al., 2015), and changes in the expression of BDNF and 5-HT in the prefrontal cortex have been implicated in the pathophysiology of depression (Gibney et al., 2013; Yu et al., 2015) and the therapeutic effect of antidepressants (Engel et al., 2013).

In this study, an animal model of isolated TPOAb-positive mice was established to explore whether TPOAb could cause post-partum depressive-like behaviors, and the expression of BDNF and 5-HT levels in the prefrontal cortex were evaluated using behavioral, molecular, and proteomic methods.

## Materials and Methods

### Animals

We obtained eighty 4-week-old female C57BL/6 mice from the SLAC Laboratory Animal Co. Ltd. (Shanghai, China). All animals and experiments were approved by the Animal Care and Use Committee of China Medical University, which complies with the National Institutes of Health Guide for the Care and Use of Laboratory Animals. All mice were individually housed on a 12/12 h light/dark schedule at 24 ± 1°C with free access to food and water except where noted. All animals were randomly assigned to two groups: the TPOAb-positive group (T group, *n* = 60) and the control group (C group, *n* = 20).

**FIGURE 1:**
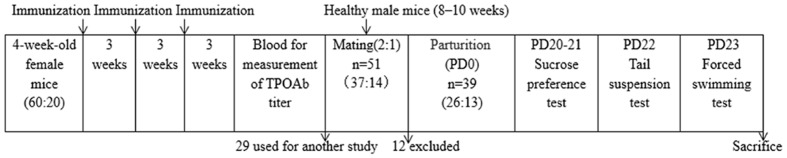
**Schematic of the experimental timeline and the number of animals at different time points.** PD, post-partum day.

Briefly, the mice in the T group were immunized by injection of the adenovirus encoding the full-length cDNA of mTPO (mTPO-Ad), at 2.0 × 10^10^ PFU per injection, in the thigh muscle (Genechem Technology Co. Ltd. Shanghai, China) every 3 weeks for a total of three times. The mice in the control group were immunized with the empty adenovirus vector instead. After three rounds of immunization, venous blood was taken through the inner canthal orbital vein and centrifuged, followed by the measurement of the TPOAb titer. A total of 51 females were used for the rest of our study after the exclusion of 29, which were used for another study. Two mice in both groups were mated with a single C56BL/6 male mouse (aged 8–10 weeks). The day of birth was designated post-partum day 0 (PD0). Of the 51 females, six did not become pregnant, four had other extraneous health issues, and two died without definitive reasons; thus, only 39 mice (T group: C group = 2:1) were studied further. On PD20 (i.e., weaning), which in some respects is similar to the post-partum period in humans (1–3 months after birth; Davis et al., 2010), animals were subjected to the following behavioral tests and then sacrificed for examination of the maternal brain. The timeline for the experiments is shown in **Figure 1**.

### Behavioral Testing

Behavioral tests began at PD20 under dim light and low noise levels. The behavior of mice in the tail suspension test (TST) and forced swimming test (FST), including the immobility time, was monitored for 6 min by a video camera and scored by two trained observers blinded to the group assignment.

#### Sucrose Preference Test (SPT)

Animals were caged individually and first trained to consume a 1% (w/v) sucrose solution for 12 h. After another 12 h period of food and water deprivation, the mice were allowed to choose between a 1% (w/v) sucrose solution and distilled water, which were placed at the same height, randomly on the left or right sides of the cages, for the next 12 h. Both bottles were weighed and recorded every 2 h for the calculation of consumption. SP (%) was calculated by dividing sucrose solution intake by total liquid intake (sucrose solution + distilled water intake) and taken as the sensitivity to reward (First et al., 2013).

#### Tail Suspension Test (TST)

The TST was carried out as described by Babri et al. (2014), with slight modification. One by one, the mice were suspended from their tails 1 cm from the end using medical adhesive tape. The heads of the mice were approximately 30 cm above the floor. Every mouse was recorded for 6 min, of which the first 2 min were for acclimatization, and the time spent immobile during the last 4 min of the testing period was measured. The time spent immobile was defined as a lack of all bodily movement except for whisker movement and respiration. The mice that climbed up their tails were removed from the analysis.

#### Forced Swimming Test (FST)

The FST was conducted as described by Babri et al. (2014). Briefly, each mouse was placed individually in a transparent cylinder (diameter: 13 cm; height: 20 cm) containing 12 cm of water maintained at 25 ± 1°C. The water was changed, and the cylinders were cleaned every testing session. All mice were tested for 6 min, dried gently with towels, and then returned to their home cages. The duration of immobility was evaluated during the last 4 min of the test after acclimatization for 2 min. Each mouse was judged to be immobile when it remained floating without struggling in the water, making no movements other than those necessary to keep its head above water.

### Measurement of Serum TPOAb and Free Thyroxine (FT4)

The blood was taken from the inner canthus venous plexus and stored at -80°C after centrifugation. Murine serum TPOAb levels were analyzed by ELISA. Here, 1 μg/mL of mTPO antigen (Cloud-Clone Corp., Arg636-Leu832, USA) was coated on 96-well plates overnight at 4°C, followed by washing, blocking with 1% bovine serum albumin (BSA)/PBS, and incubated with individual murine serum (dilution = 1:100). After the incubation with the secondary antibodies (alkaline phosphatase-labeled rabbit anti-mouse IgG; Sigma, A9044, USA, 1:40,000 dilution), TMB (Sigma, T0440, USA) was added. At the appropriate time, 2 M of HCl was added to terminate the reaction. Absorbance was measured using the 450-nm filter (TECAN, F200PRO, Switzerland). Since the TPOAb standard is unavailable, the OD value was used for comparison.

Murine serum FT4 was measured at a 1:5 dilution in duplicate wells using a commercial ELISA kit (Cloud-Clone Corp., CEA185Ge, USA; Hu et al., 2016). All experimental steps were performed according to the kit specifications. The minimum detectable dose of FT4 is typically 0.48 pg/mL. The intra-assay CV was <10%, and the inter-assay CV was <12%.

### Prefrontal Cortex Total Thyroxine (TT4)

After cardiac perfusion with normal saline, bilateral tissue punches of the prefrontal cortex were rapidly separated from the brain sample by hand dissection using a stereomicroscope and a thin brush as described by Jia et al. (2015), quickly frozen in liquid nitrogen, and then stored at –80°C. The prefrontal cortex homogenate, which was homogenized in 500 μl of phosphate-buffered saline solution, was used as the working solution, followed by detection with another commercial ELISA kit (Cloud-Clone Corp., CEA452Ge, USA; Hu et al., 2016). All samples were tested in duplicates according to the kit instruction manual. The minimum detectable dose of TT4 is typically 1.42 ng/mL. The intra-assay CV was <10%, and the inter-assay CV was <12%.

### Real-Time PCR

The total RNA from the prefrontal cortex was extracted using TRIzol (Invitrogen, 15596-026, USA) according to the standard procedure. After the determination of the RNA concentration and purity with Nano Drop2000C (Thermo Scientific, USA), the total RNA (1 μg) was reverse-transcribed with random primers using the Reverse Transcription Reagent Kit (TaKaRa, RR036A, Japan) in accordance with the manufacturer’s protocol. All amplification reactions were performed on the ABI PRISM 7500 sequence detection system (Applied Biosystems, USA). For quantitative real-time PCR, 25 ng of cDNA was used, and single transcript levels of genes were detected using a QuantiTect SYBR green master mix (TaKaRa, RR820A, Japan). Primers used to detect synaptic transcripts were designed (Sangon Biotech, China) as follows: BDNF forward (5′-ATC GGT TCA CAG GAG ACA T-3′) and reverse (5′-TCA GGT CAA CAT AAA CCA CCA-3′) and GAPDH forward (5′-TGT GTC CGT CGT GGA TCT GA-3′) and reverse (5′-TTG CTG TTG AAG TCG CAG GAG-3′). For each sample, gene expression was determined by normalizing against the expression of the GAPDH gene. Data were analyzed using the comparative CT method to correct for the amplification efficiency. All samples were measured twice.

### Western Blotting Analysis

Western blot procedures were conducted as described by Zhang et al. (2015). The prefrontal cortex protein was extracted with the Total Protein Extraction Kit (KeyGen Biotech, KGP2100, China), followed by the measurement of the protein concentration using the BCA Protein Assay Reagent Kit (Beyotime, P0012S, China). Reagents included SDS-PAGE loading buffer (Beyotime, P0015, China), PageRuler^TM^ Prestained Protein Ladder (Thermo Scientific, 26616, USA), rabbit polyclonal anti-BDNF (Merck-Millipore, AB1534SP, Germany, 1:500 dilution), mouse monoclonal anti-GAPDH (Zhongshan Golden Bridge, TA-08, China, 1:1,000 dilution), HRP-conjugated goat anti-rabbit IgG (h + l; Immunology Consultants Laboratory, Inc., GGHL-15P, USA, 1:5,000), HRP-conjugated goat anti-mouse IgG (Zhongshan Golden Bridge, ZB2305, China, 1:1,000 dilution), and Pierce^TM^ ECL Western Blotting Substrate (Thermo Scientific, 32209, USA). Images were processed and analyzed using ImageJ software (National Institutes of Health, USA).

### Analysis of 5-HT Levels by UHPLC–MS/MS

The UHPLC–MS/MS method was developed to determine the concentrations of neurotransmitters (Gonzalez et al., 2011) with higher peak capacity, greater resolution, and higher speed. For 5-HT detection, the tissue samples were prepared on ice and precisely weighed, followed by homogenization in 200 μl of 0.2% formic acid solution with a glass homogenizer. Standard products (Fluorophen Ltd., 50-67-9, UK) were prepared at a concentration of 400 μg/ml with methanol water (7:3, W/W) used as the standard stock solution and further diluted with methanol to obtain working standards. The mobile phase consisted of 0.1% formic acid water solution: acetonitrile (95:9, W/W). The auto sampler was conditioned at 4°C, the flow rate was 0.3 ml/min, and the injection volume was 10 μl. An external standard curve was run on the day of analysis. The amount of 5-HT in the prefrontal cortex was expressed in μg/mg tissue.

### Statistical Analysis

All data were statistically analyzed using SPSS 21.0 software (IBM Corp.). Data are expressed as the mean ± standard error of measurement (SEM), and *p* < 0.05 was considered statistically significant. The *t*-test was used to assess the statistical significance of the differences between the two groups. The correlation analysis was performed by Pearson’s Correlation test after normal analysis.

## Results

### Assessment of Serum TPOAb Titer and Thyroid Hormone

**FIGURE 2:**
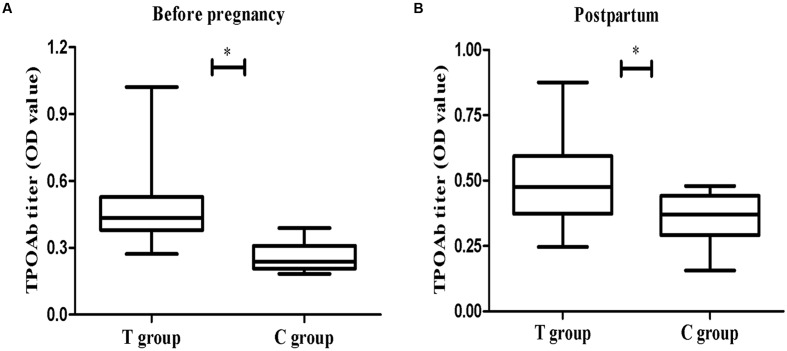
**The concentration of thyroid peroxidase antibodie (TPOAb) in serum at different time points.** **(A)** The left panel shows the measurements taken before pregnancy (T group, *n* = 49; C group, *n* = 20). **(B)** The right panel shows the measurements taken on PD23 (T group, *n* = 37; C group, *n* = 14). The data are shown as the mean OD ± SEM. ^∗^*p* < 0.05 for the T group vs. the C group (independent sample *t*-test).

As shown in **Figure 2A**, the serum TPOAb measured before pregnancy in the T group was significantly higher than in the C group (*p* < 0.01). There was no difference in FT4 levels between the two groups (**Table 1**). Serum TPOAb levels measured post-partum (after behavior tests) were still higher in the T group than in the C group (*p* < 0.05, **Figure 2B**). No change was observed in the prefrontal cortex TT4 concentration, indicating that mTPO-Ad did not influence local thyroid hormone levels (**Table 1**).

**Table 1:** Concentration of FT4 in serum before pregnancy and TT4 in the prefrontal cortex on PD23.

	Serum FT4 (pg/ml) Before pregnancy (n)	Prefrontal cortex TT4 (ng/ml) PD23 (n)
T group	1.986 ± 0.035 (45)	3.937 ± 0.238 (12)
C group	1.87 ± 0.054 (10)	4.814 ± 0.428 (6)


*Values are expressed as the mean ± SEM. The results suggested that no differences in the concentrations in the serum or prefrontal cortex existed between the two groups, *p* > 0.05.*

### Behavioral Results

#### Sucrose Preference Test (SPT)

**FIGURE 3:**
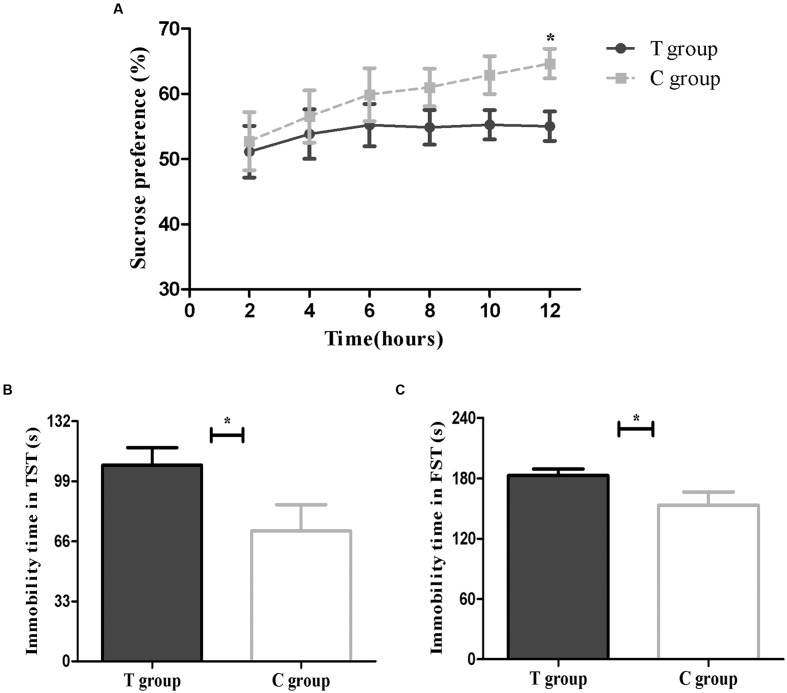
**Behavioral results are shown above.** **(A)** Sucrose preference test (SPT; T group, *n* = 26; C group, *n* = 13). **(B)** Tail suspension test (TST; T group, *n* = 22; C group, *n* = 12). **(C)** Forced swimming test (FST; T group, *n* = 26; C group, *n* = 13). The data are shown as the mean ± SEM. ^∗^*p* < 0.05 for the T group vs. the C group (independent sample *t*-test).

Depression-like behavior was defined as a decrease in SP (%), i.e., the percentage of sucrose consumption. The T group showed less relative sucrose consumption than the C group at all time points (2, 4, 6, 8, 10, and 12 h), although this difference was only significant at 12 h (*p* < 0.05; **Figure 3A**). The significant reduction in SP (%) suggested that elevated TPOAb might increase behavioral anhedonia post-partum.

#### Tail Suspension Test (TST)

The increase in time spent immobile during the TST was also defined as depression-like behavior. The results showed that the mice in the T group had a significantly longer immobility time than the C group (*p* < 0.05; **Figure 3B**), which indicated that the T group might be exhibiting behavioral despair.

#### Forced Swimming Test (FST)

The FST was another method to evaluate behavioral despair. As shown in **Figure 3C**, we found that mice in the T group had a longer immobility time in the FST than mice in the C group (*p* < 0.05), consistent with the TST results.

### BDNF mRNA and Protein Expression and 5-HT Concentrations in the Prefrontal Cortex

**FIGURE 4:**
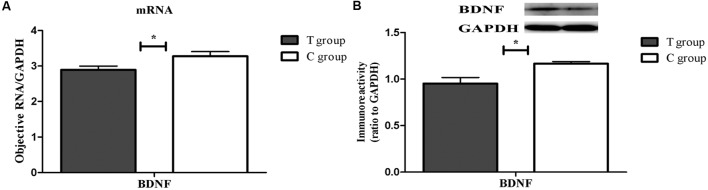
**The expression of brain-derived neurotrophic factor (BDNF) mRNA and protein in the TPOAb-positive group and the control group in the prefrontal cortex on PD23.** Each bar represents the mean ± SEM, ^∗^*p* < 0.05. *n* = 8 per group. **(A)** BDNF gene expression was normalized to the reference gene GAPDH. **(B)** The bands depict representative findings in the C and T groups. BDNF was normalized to the reference protein GAPDH, and the bar graphs show the results of the semi-quantitative measurement.

**FIGURE 5:**
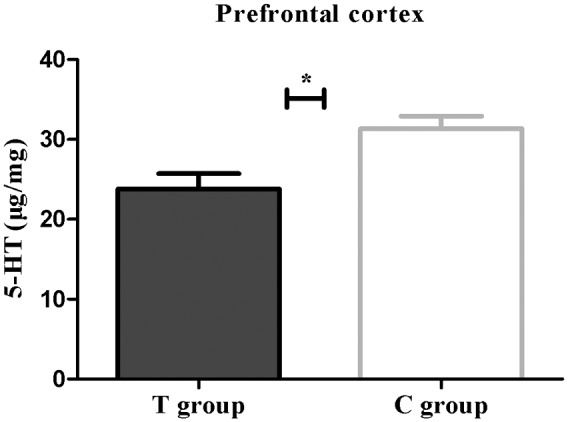
**The 5-HT concentration (μg/mg) expressed as the mean ± SEM.** *n* = 6 per group. ^∗^*p* < 0.05 for the T group vs. the C group.

Both real-time PCR and Western blot analysis indicated that BDNF mRNA and protein expression were both significantly lower in the T group than that in the C group (*p* < 0.05; **Figure 4**). UHPLC–MS/MS analysis showed that the 5-HT concentration in the prefrontal cortex in the T group, which showed depressive behavior, was significantly lower than that in the C group (*p* < 0.05; **Figure 5**). These findings support the hypothesis that prefrontal cortex BDNF and 5-HT are involved in PPD (Suda et al., 2008; Haim et al., 2016).

## Discussion

In this study, we established the isolated TPOAb-positive model and found that elevated TPOAb before pregnancy resulted in depressive behaviors post-partum as evidenced by significantly lower SP at the 12-h time point and a longer immobility time in the FST and TST. In addition, these behavioral changes were accompanied by lower BDNF and 5-HT levels in the prefrontal cortex but no change in TT4 levels. These experiments provided further evidence for the association between TPOAb and PPD in an isolated TPOAb-positive mouse model, which was consistent with the clinical studies mentioned above (Kuijpens et al., 2001; Albacar et al., 2010; Le Donne et al., 2012).

Approximately 10–20% of women in the first 3 months of pregnancy were positive for TPOAb or TgAb with normal thyroid hormone levels (Stagnaro-Green et al., 2011). Kuijpens et al. (2001) first found TPOAb to be independently associated with depression at 12 weeks gestation and at 4 and 12 weeks post-partum. A later, larger prospective follow-up study that enrolled 1,017 pregnant women from the general population indicated that TPOAb was related to major depression at 12 and 24 weeks of gestation but not at 36 weeks, when the downregulation of the immune system reached its maximum, and the occurrence of major depression declined with decreasing TPOAb titers throughout pregnancy (Pop et al., 2006). There was also evidence that the TPOAb titer was significantly higher in women at risk for PPD than those not at risk for PPD using three evaluation scales (Le Donne et al., 2012; Groer and Vaughan, 2013). However, in different study populations, other researchers found no association between TPOAb and depression (Engum et al., 2005; Albacar et al., 2010; Delitala et al., 2016), even during 1 week post-partum (Lambrinoudaki et al., 2010). Because the results in studies on the association between TPOAb and depression in the clinical literature are always partially mixed due to thyroid hormone levels or other social factors, in the current study, an isolated TPOAb-positive animal model was established to exclude probable confounding factors.

The establishment of animal models with elevated TgAb or TPOAb is an effective approach to investigate the mechanism of TgAb or TPOAb-related disease. Unlike TgAb, the TPOAb-positive animal model was developed only within the past 12 years, and the scientific community has not yet formed a consensus about it (Ng et al., 2004). Unlike the method of H.P.NG et al., a TPOAb-positive animal model was established by immunizing C57BL/6 mice with mTPO-Ad, the spatial epitope instead of the linear epitope of TPO, using a method developed in our lab, which successfully induced elevated mTPOAb concentrations that lasted into the post-partum period. The use of mTPO might be roughly the same as its effects *in situ*. Therefore, here, mTPOAb was induced *in vivo* to facilitate the study of TPOAb and depressive behavior. Although there have been studies that have focused on the mechanism of the association between thyroid abnormalities and depression (Ge et al., 2014; Yu et al., 2015), the present study is the first to report the impact of TPOAb on PPD-like behaviors and the underlying mechanism in mice.

Most studies use SPT, FST, or TST in laboratory animals (Babri et al., 2014; Fernandez et al., 2014; Ge et al., 2014) to characterize PPD-related behavior. However, a single behavioral disturbance is likely not sufficient (Anisman and Matheson, 2005), and a wide divergence may exist in different depressive-like behaviors (Fernandez et al., 2014). In our present study, the TPOAb-positive group displayed depressive behavior as indicated by significant decreases in the SP at the 12-h time point and by increases in immobility during the FSTs and TSTs, which are in agreement with the clinical conclusions. Among the three depressive behaviors, a Pearson’s correlation test showed that only the immobility time during the TST correlated with TPOAb titer post-partum (*p* < 0.01, *r* = 0.556) or before pregnancy (*p* < 0.05, *r* = 0.439) in the T group, indicating that the TST may be a more sensitive indicator in this animal model.

The prefrontal cortex is a brain region which has been linked to cognitive behavior, personality expression, and social behavior, including PPD (Moses-Kolko et al., 2010; Haim et al., 2016; Palmfeldt et al., 2016), and is important in the development and treatment of depression (Koenigs and Grafman, 2009). Local thyroid hormone deficiency was noted prior to the reduction in plasma and was found to be related to depressive behaviors (Ge et al., 2014). In our study, TT4 levels were measured in the prefrontal cortex, which might change prior to serum thyroid hormone levels (Ge et al., 2014). The results showed that there was no difference between the two groups, indicating that the depressive behavior in the T group had nothing to do with differences in local thyroid hormones.

Brain-derived neurotrophic factor, a member of the neurotrophin family, is a small basic protein that supports the survival of neurons by playing critical roles in cell differentiation, neuronal survival, migration, and synaptic plasticity and has been suggested to be involved in the pathogenesis of depression (Kunugi et al., 2010). BDNF deficiency is thought to partially contribute to the reduced synaptic plasticity and neuronal atrophy seen in depressive patients (Jacobs et al., 2000; Jaako-Movits and Zharkovsky, 2005). Chronic stress or antidepressive drugs may also attenuate or enhance synaptic plasticity in the prefrontal cortex via alteration of BDNF expression and function. Suda et al. (2008) established an animal model of PPD to mimic human pregnancy and the post-partum period, which also revealed that decreased BDNF levels could contribute to depressive behavior. 5-HT, a monoaminergic neuromodulator, is also confirmed to help relieve depressive symptoms by mediating neuroplastic events. Antidepressant-induced elevation of 5-HT levels may induce structural modifications including neuroplasticity, neurogenesis and other adaptive changes even in the post-partum prefrontal cortex (Haim et al., 2016). The characterization of 5-HT–BDNF interactions has also been reviewed (Homberg et al., 2014); the increase in 5-HT induced by 5-HT reuptake inhibitors upregulates BDNF expression (Deltheil et al., 2008). Here, we hypothesize that the data indicating that the 5-HT and BDNF levels in the prefrontal cortex were significantly lower in the T group than those in the C group were consistent with the findings of previous studies (Homberg et al., 2014; Yu et al., 2015). Hence, the depressive behavior observed in the T group may be due to changes in both 5-HT and BDNF in the prefrontal cortex.

There is already evidence linking depression to proinflammatory cytokines in patients with autoimmune disease (Postal and Appenzeller, 2015). Proinflammation can cause increased indoleamine-2,3-dioxygenase (IDO), which is associated with inflammatory processes and serotonergic systems (Xu et al., 2015); however, the concentration of proinflammatory cytokines was not measured in the present study. The study only employed the TST, FST, and SP to characterize PPD but did not involve other maternal care behavior tests, such as nursing, grooming/licking pups, or manipulating nest bedding. In addition, since the serum TPOAb measured in the T group was significantly higher than in the C group not only before pregnancy but also post-partum, we cannot determine whether the higher TPOAb titer could increase the risk of depression and reduce the levels of BDNF and 5-HT before pregnancy. We will focus on these limitations in the future.

In summary, to our knowledge, this is the first concrete demonstration of the association between isolated TPOAb and PPD in an animal model. These findings suggest that women positive for TPOAb might be at a higher risk of PPD. Decreases in BDNF expression and 5-HT concentration in the prefrontal cortex could be involved in the mechanism of PPD.

## Author Contributions

The conception and design of the work: YyiZ, XW, ZS, YhZ, AL, TZ, YyuZ, and WT. The acquisition, analysis, or interpretation of data for the work: YyiZ, XW and ZS. Performed the experiments: YyiZ and XW. Drafting the work: YyiZ. Revising the work critically: ZS. Final approval of the version to be published: ZS and WT.

## Conflict of Interest Statement

The authors declare that the research was conducted in the absence of any commercial or financial relationships that could be construed as a potential conflict of interest.

The reviewer JC and handling Editor declared their shared affiliation, and the handling Editor states that the process nevertheless met the standards of a fair and objective review.
